# Erosion of Gene Co-expression Networks Reveal Deregulation of Immune System Processes in Late-Onset Alzheimer’s Disease

**DOI:** 10.3389/fnins.2020.00228

**Published:** 2020-03-20

**Authors:** John Stephen Malamon, Andres Kriete

**Affiliations:** Bossone Research Center, School of Biomedical Engineering, Science and Health Systems, Drexel University, Philadelphia, PA, United States

**Keywords:** Alzheimer’s, networks, immune system, synapses, functional, eQTL, WGCNA

## Abstract

We have applied a novel and integrative analysis framework for next-generation sequencing (NGS) data to 503 human subjects provided by the Religious Orders Study and Memory and Aging Project (ROSMAP) to examine changes in transcriptomic organization and common variants in association with late-onset Alzheimer’s disease (LOAD). Our framework identified seven reproducible, co-regulated modules after quality control (QC), clinical segregation, preservation filtering, and functional ontology analysis. These modules were specifically enriched in several innate and adaptive immune system processes, the synaptic vesicle cycle, and Hippo signaling. Topological and functional erosion of these modules due to shedding of genes and loss of in-module connectivity was diagnostic of disease progression. Perturbation analysis revealed that only 1% of eQTLs overlapped genes participating in these co-regulated modules. Common variants nevertheless identified components of the immune systems like human leukocyte antigen (HLA) complex and microtubule-associated protein tau (MAPT) regions in association with LOAD. Our results implicate microglial function, adaptive immune response, and the structural degeneration of neurons as contributors to the transcriptional deregulation observed along with common genetic variants in the progression of LOAD.

## Introduction

Late-onset Alzheimer’s disease (LOAD) is a complex condition involving tau protein aggregates or tauopathy, amyloid and lipid processing, aging, immune system response, metabolism, lysosomal processing, and cerebrovascular health (Rogers et al., 1988; Braak et al., 2011; Jevtic et al., 2017; Wang et al., 2017). Progress in understanding and describing this large and diverse set of biological systems is in part determined by our ability to fully integrate clinical neuropathological data with comprehensive models that combine several modes of next-generation sequencing (NGS) data. To this end, we have applied our novel and integrative analysis framework to 503 subjects (305 cases/198 controls) provided by the Religious Orders Study and Memory and Aging Project (ROSMAP) study (Bennett et al., 2012) to develop a detailed landscape of the genetic and regulatory systems involved in LOAD, specifically with respect to clinical scores. Our study accomplished the following four objectives: (1) identified changes in transcriptional organization in association with clinical phenotypes; (2) characterized the systematic transcriptomic and functional changes accompanying LOAD through clinical segregation co-expression analysis; (3) identified common genomic loci involved in LOAD; and (4) tested the relationship between predicted expression quantitative trait loci (eQTLs) and systematic changes in gene expression.

One of the core elements of our approach is a weighted gene co-expression analysis (WGCNA), enabling the classification and identification of highly correlated and connected modules of genes grouped by co-expression (Zhang and Horvath, 2005; Langfelder and Horvath, 2008). Network modules can be described as series of interrelated nodes and edges. Here, nodes are messenger RNA (mRNA) transcripts. Edges represent the correlation coefficients between two or more given nodes, where degrees are the number of edges shared by nodes. Genes usually have many regulators, so we chose a hierarchical co-expression model. Our combined approach increases specificity by reducing large co-expression networks to only functionally significant and highly reproducible modules. Functional significance is defined as the Gene Ontology biological process *p*-value and reproducibility is defined as the module preservation Z-score (Langfelder et al., 2011). Co-expression analysis has been successfully applied in Alzheimer’s disease (AD), incorporating clinical scores and differential expression to identify co-regulated modules changing with disease in an “all-in-one” analytical design (Miller et al., 2008; Liang et al., 2018; Meng and Mei, 2019). However, the common approach to co-expression modeling does not include clinical segregation analysis. Here we provide clinical segregation for three groups: no cognitive impairment (NCI), mild cognitive impairment (MCI), and AD subjects. Additionally, strong genetic associations have been observed in LOAD (Naj et al., 2011; Lambert et al., 2013; Sims et al., 2017) along with systematic changes in gene expression profiles and transcriptional organization (Miller et al., 2008, 2010; Zhang et al., 2013; Ramasamy et al., 2014). Therefore, we hypothesize that genetic variation should account for changes in gene expression observed in transcriptomic analyses. We systematically tested the relationship between predicted eQTLs and transcriptomic organization to show underlying perturbations in gene networks that can partially account for changes observed in co-expression analyses.

## Methods

The Accelerating Medicines Partnership (AMP) provides a variety of multi-platform next-generation sequence (NGS), clinical, and other –omics data. We selected all subjects from the ROSMAP study with overlapping clinical, RNA-seq, and DNA-seq data from the prefrontal cortex from a total of 503 elderly individuals varying from cognitively healthy to diagnosed AD (Bennett et al., 2012; De Jager et al., 2018). All subjects reported race as Caucasian. According to study details, RNA was extracted from the gray matter of the dorsolateral prefrontal cortex and quantified using the NanoDrop spectrophotometer.;101-bp paired-end, Illumina HiSeq reads were aligned to the human reference genome 19 (hg19). Genotype data were generated using the Affymetrix GeneChip 6.0 platform and filtered based on the following quality control (QC) criteria: genotype call rate less than 99%, minor allele frequency (MAF) less than 2%, and a Hardy–Weinberg equilibrium threshold below 1%. A total of 619,377 single nucleotide polymorphisms (SNPs) passed QC and were used in this analysis.

### Analysis Framework

Our analytical framework was previously introduced (Malamon and Kriete, 2018) and extended here to include additional features, such as clinical segregation, module preservation, gene set, and functional enrichment analyses (see Supplementary Figure 1 for workflow diagram and full description of methods). This workflow consists of four main components: QC, co-expression modeling, functional enrichment, and eQTL analysis. We perform a comprehensive, three-tiered QC process to normalize and reduce the RNA-seq dataset to the 20,000 most informationally dense and connected transcripts. Co-expression networks are constructed using the WGCNA toolkit (Langfelder and Horvath, 2008). Next, we apply WGCNA’s module preservation testing procedure to measure statistical reproducibility in all modules. We exclude all modules with preservation Z-scores below 10 standard deviations. Higher Z-scores signify modules that reoccur despite changing input conditions. These become candidate modules. Functional term and enrichment analyses are performed on all candidate modules. Gene set enrichment analysis (GSEA) (Subramanian et al., 2005) was used to examine larger functional network trends and reproduce candidate modules and genes. We provide a novel approach leveraging clinical segregation co-expression analysis to examine and compare alterations in network and module structure and organization with disease progression. For segregation analysis, QC, co-expression modeling, and functional enrichment were repeated for all clinical subgroups. Finally, genome-wide association (GWA) and perturbation analyses were performed. GWA provides all genomic loci (SNPs) predicted in association with disease status. eQTL analysis provides the predicted effects of SNPs on gene expression. Perturbation analysis was performed by overlapping co-expression module genes with eQTLs.

### Clinical Segregation Analysis

**FIGURE 1:**
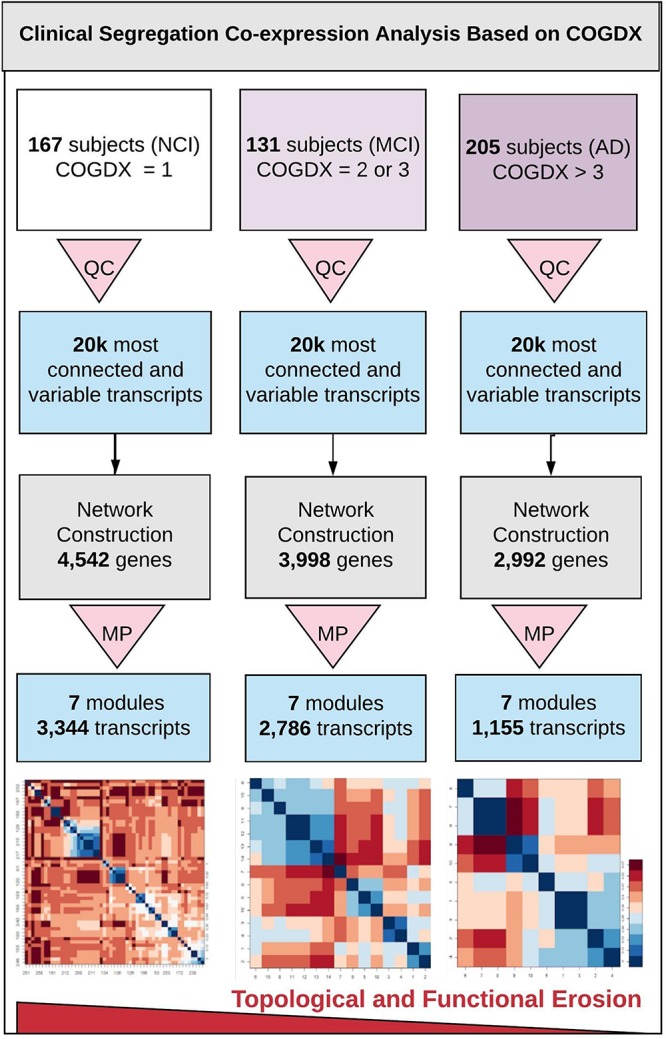
Overview of clinical segregation co-expression analysis. An outline of our novel approach for independently analyzing and comparing co-expression networks and module characteristics in regard of clinical disease progression scores. The three vertical lanes represent COGDX segregation for three different cognitive scores (NCI, MCI, and AD) as defined in Supplementary Table 1. All subgroups were processed independently. First, quality control (QC) was applied to each set to retain only the 20,000 most informationally dense and variable transcripts. Next, networks were constructed with identical modeling parameters for all three subgroups. Module preservation (MP) testing was used to filter modules to only those that were highly reproducible (Z-score > 10), leaving seven modules. Within these modules, we observed a significant loss in the total transcripts classified, within-module connectivity, and functional term enrichment in association with disease progression. Heatmap tiles in the bottom lane refer to functionally significant GO biological process terms.

Figure 1 outlines our clinical segregation protocol, which was designed to assess how transcriptomic differences are presented in clinical subgroups. We segregated samples by extracting sample data based on COGDX and CERAD scores and processing each group independently in WGCNA (see Supplementary Table 1 for clinical definitions and Supplementary Figure 2 for data plot). COGDX collapses 19 different neuropsychological tests into a single “Global Cognitive Score” (De Jager et al., 2018). The CERAD protocol provides neuropathological classifications for disease based on a wide variety of life-style, neuropsychological, and cognitive tests (Mirra et al., 1991). For COGDX, we segregated samples leaving 167 subjects with NCI, 131 subjects with MCI, and 205 subjects with an Alzheimer’s diagnosis (AD). For CERAD, we segregated samples leaving 130 subjects with no AD (CERAD_1), 226 subjects with possible or probable AD (CERAD_2), and 147 subjects with confirmed AD (CERAD_3). We independently processed and analyzed all six clinical subgroups using WGCNA with the same network parameters for all experiments.

## Results

### Network Construction

We calculated the transcriptomic network’s total connectivity using the median-based bi-weight mid-correlation, which is more accurate than Pearson’s method for gene co-expression modeling (Zheng et al., 2014). Raising the soft-threshold to a power of 6 produced an overall R^2^ value of 0.895, as seen in Supplementary Figure 3. Note that the *R*^2^ value rises sharply and quickly flattens out with a slope of −1.080 at just six iterations. WGCNA was used to construct the initial co-expression network (Supplementary Figure 4) using all 503 subjects. Careful consideration was used in selecting the criteria for module identification, also known as branch trimming. We identified 26 distinct modules, totaling 4,429 transcripts with an average of 201 genes per module. Modules contain directionally signed groups of genes. In other words, genes in the same module are co-expressed in the same direction and well correlated with one another. We selected to lean on the side of specificity by not partitioning around medoids, leaving a total of 15,571 (77.85%) transcripts out of modules (unclassified), as indicated in gray (Supplementary Figure 4). Overall, the dendrogram shows clean, distinct clustering with sufficient levels of local dissimilarity. WGCNA arbitrarily assigns module names by color, i.e., gray and magenta. Supplementary Figure 5 shows all module-to-module and module-to-eigentrait (eigenvector of clinical metric) correlations for each of the four clinical NP traits. Supplementary Spreadsheet S1 contains all co-expressed genes grouped by module.

### Clinical Segregation and Module Preservation Analysis

To investigate network and module characteristics with respect to disease progression, we segregated samples according to COGDX and CERAD scores and analyzed each of the six clinical subgroups independently in WGCNA. Clinical subgroups were assigned according to Supplementary Table 1. For example, subjects with COGDX scores of 0 or 1 were assigned to the NCI group. For COGDX, we uniquely classified 4,542, 3,998, and 2,992 genes for the NCI, MCI, and AD groups, respectively. For CERAD, we uniquely classified 3,426, 3,957, and 3,991 genes for the CERAD_1, CERAD_2, and CERAD_3 groups, respectively. WGCNA’s *module preservation* function allowed us to accurately measure module reproducibility through permutation testing. We calculated module preservation Z-statistics using 200 permutations for all six subgroups. See Supplementary Figure 6 for preservation statistics. Modules with Z-scores above 10 are not obtained by random chance and can be reliably reproduced (Langfelder et al., 2011; Li et al., 2015). A total of seven candidate modules (Table 1) survived preservation testing. Segregation by COGDX showed increased reproducibility and stability in module preservation over segregation based on CERAD assessment scores; therefore, we selected COGDX modules for further analysis.

**TABLE 1:** Statistically significant functional terms for seven well-preserved modules sorted by adjusted *p*-value.

**Module name**	**# of genes**			**Highest fold enrichment ontology term**	**Fold enrichment**	**Lowest *p*-value**	**Adjusted *p*-value**
	**NCI**	**MCI**	**AD**				
Magenta	191	145	99	Regulation of T cell activation via T cell receptor contact with antigen bound to MHC molecule on antigen presenting cell	>100	Immune system process	1.80E-40
Yellow	291	217	27	Regulation of synaptic vesicle cycle	12.67	Modulation of chemical synaptic transmission	1.32E-14
Blue	859	693	222	Hippo signaling	9.74	Regulation of cell signaling	2.69E-14
Turquoise	1305	992	429	Regulation of complement activation	6.26	Cellular component organization or biogenesis	1.47E-08
Green	66	168	54	Phospholipid dephosphorylation	12.64	Cellular protein metabolic process	1.67E-07
Red	181	65	129	Regulation of synapse organization	5.23	Chemical synaptic transmission	5.11E-05
Brown	451	506	195	Regulation of complement activation	8.86	Humoral immune response	1.60E-04

*NCI, MCI, and AD columns provide the number of module genes by COGDX subgroup. GO’s DAVID functional analysis results are provided for seven modules. Highest fold enrichment and lowest *p*-value (Bonferroni adjusted) are provided in the fifth and seventh columns with the predicted biological process.*

### Functional Enrichment of Co-expression Modules

Biologically relevant, functional pathways should be reproducible and overlap known LOAD pathologies. To this end, we queried the GO database to examine the functional ontologies of the seven candidate modules. Table 1 provides statistically significant biological process terms involving known LOAD pathologies. The “magenta” module, which showed the strongest functional association, was well-correlated with COGDX and highly enriched with many immune-related genes including ABI3, APBB1IP, CD33, CD86, DOCK2, human leukocyte antigen (HLA)-DRA, HLA-DMB, MS4A4A, MS4A6A, MS4A7A, TREM2, and TYROBP. Other “magenta” GO terms include “complement pathway,” “cytokine signaling,” “neutrophil degranulation,” and “Toll-Like receptor activation.” Additional modules involving the immune system included the “turquoise” and “brown” modules, which were both enriched for the “regulation of complement activation.” A GO “cellular components” query revealed the “dendrite membrane” as significant for the “yellow” module (*p*-value = 3.73E-06). This observation is consistent with the “biological process” query results, which provided several synaptic processes including “neuronal projection,” “vesicle cycle,” and “synaptic maintenance.” The “blue” module was functionally enriched for genes in the Hippo signaling pathway, including AMOT, FAT4, LAT2, TJP1, TJP2, STK3, and YAP1. “Fatty acid oxidation” was also significant for the “blue” module. Additionally, cell-specific enrichment was performed on all seven modules (Uhlen et al., 2015; Kuleshov et al., 2016; Lachmann et al., 2018). See Supplementary Table 2 for results.

### Organizational Changes in Immune Module

**FIGURE 2:**
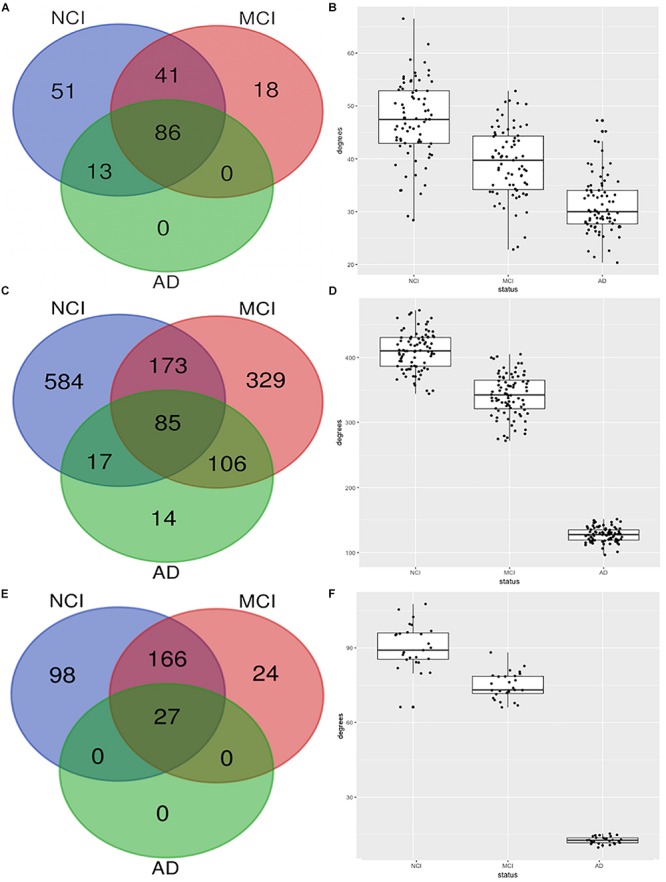
Erosion of nodes and edges for top three functional modules by CODGX segregation. **(A)** Venn diagram of genes in the immune-enriched module (magenta) for the three COGDX subgroups, NCI, MCI, and AD. **(B)** Boxplots with the number of intramodular connections (degrees) grouped by COGDX. **(C)** Venn diagram for “blue” module (Hippo Signaling). **(D)** Boxplots of degrees grouped by COGDX for “blue” module. **(E)** Venn diagram for “yellow” module (synaptic vesicle cycle). **(F)** Boxplots of degrees grouped by COGDX for the “yellow” module.

Figure 2 shows the erosion of the “magenta” module by comparing the network characteristics of the three co-expression networks segregated by COGDX. “Magenta” contained 191, 145, and 99 genes for NCI, MCI, and AD, respectively (Figure 2A) and shared 86 genes across all clinical groups. The mean intramodular degrees for the NCI, MCI, and AD subgroups provided in Figure 2B were 47.13, 39.27, and 31.27, respectively. The “blue” module shared 85 genes in all three subgroups with 859, 693, and 222 genes, respectively. The mean intramodular degrees for each subgroup were 409.32, 341.86, and 127.42 for the “blue” module. The “yellow” module shared 27 genes with 859, 693, and 222 genes, respectively. The mean intramodular degrees for “yellow” were 90.48, 74.69, and 12.66, respectively (*p*-value = 2.2E-16). Similar trends were observed in the other four modules. ANOVA and Bartlett’s test for heteroscedasticity were performed for all transcripts by COGDX subgroup revealing a significant (*p*-value < 0.05) increase in the expression of 22 “magenta,” 31 “yellow,” and 70 “blue” genes. Heteroscedasticity was significant (*p*-value < 0.001) for 36, 48, and 74 genes, respectively. These data provide supporting evidence for the deregulation of gene networks in these three modules. Supplementary Spreadsheet S2 contains all ANOVA and Bartlett’s testing results.

### Gene Set Enrichment Analysis

The Broad Institute’s GSEA toolkit was used to identify disease-associated pathways via the KEGG biological pathway database (Kanehisa and Goto, 2000; Kanehisa et al., 2016, 2017). We performed a pre-ranked analysis with 10,000 permutations to discover differences in functional gene networks with regard to disease status. Supplementary Figure 7 provides the top and bottom five KEGG pathways sorted by *p*-value. The top five most significant (*p*-value < 1.5E-03) pathways positively enriched or under-represented in cases contained several immune-related genes also observed in co-expression modeling, including HLA-DRA, HLA-DMB, and CD86. Interestingly, cases exhibited deregulation in many immune system-related genes, which is consistent with the shedding of co-expressed genes revealed in the previous section. Negative enrichment scores denote an overrepresentation of pathway gene expression in cases. “*Alzheimer’s, Parkinson’s, and Huntington’s disease*” pathways showed high overrepresentation in cases.

### Transcription Factor Analysis

Finally, we asked whether transcription factors may be influential for the observed changes in modules. Transcription factor binding site interrogation was performed using human single-site analysis (oPOSSUM) (Ho Sui et al., 2005) carried out at 8-bit minimum specificity, 40% conservation cutoff, 5,000 bp upstream/downstream the transcription start site, 85% matrix threshold, against a background of 24,752 genes. “Magenta” genes were highly enriched (*p*-value < 0.001) for the SPI1 and Interferon Regulatory Factor 8 (IRF8) transcription factor binding motif. Genes with SP1 binding site were also enriched in genes lost from “magenta” and included CD4, CYBA, HAMP, HCST, HLA-DMA, IL18, TLR10, and TREM2. Genes with PPARG:RXRA binding site included CD4, CYBA, HAMP, HCST, HLA-DMA, IL18, TLR10, and TREM2.

### Expression Quantitative Trait Locus Analysis

**FIGURE 3:**
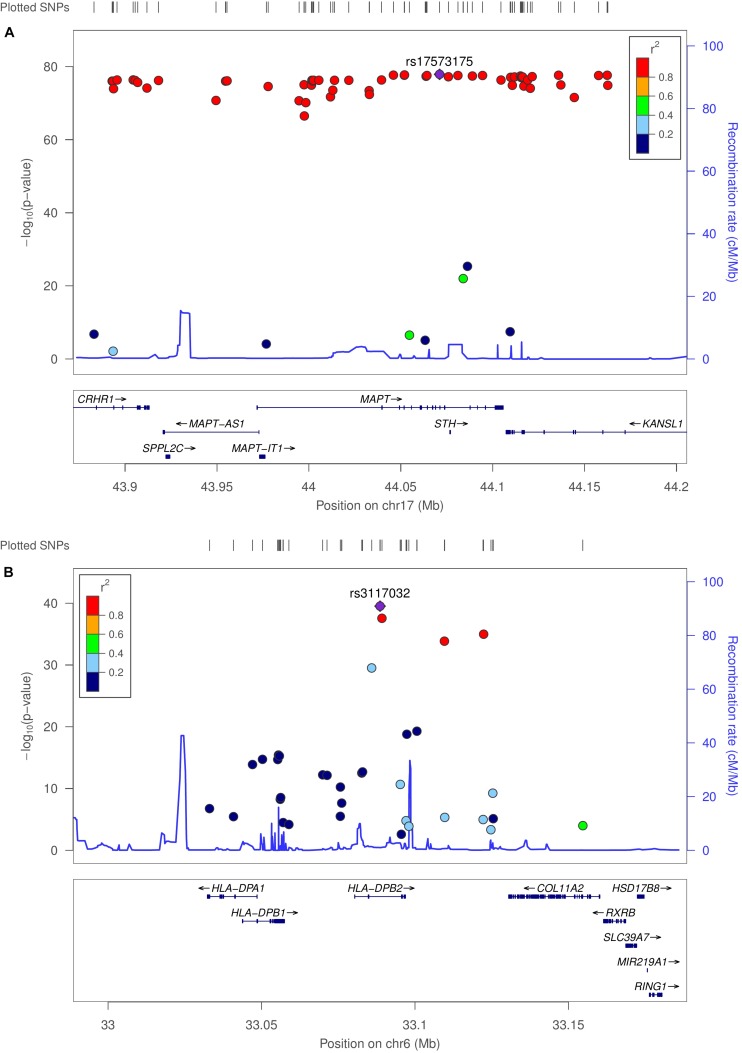
Genome-wide association plots for eQTL analysis. Regional association plots for MAPT and HLA-DPB2 regions. **(A)** Recombination rates (cM/Mb) (right vertical axis) and –log_10_ (*p*-value) (left vertical axis) for SNPs with linkage peaks in blue for the MAPT and **(B)** HLA-DPB2 regions. SNPs are colored by the linkage disequilibrium correlation coefficient (*R*^2^). Genetic linkage data were provided by the International HapMap Project.

We used MatrixEQTL (Shabalin, 2012) to test the linear associations between changes in gene expression and genotype for the same 503 individuals used in co-expression modeling. Interestingly, 90% of the top 100 eQTLs (sorted by adjusted *p*-value) occurred in the microtubule-associated protein tau (MAPT) region. Several HLA loci were statistically significant, including HLA-A, HLA-C, HLA-DOB, HLA-DP1, HLA-DRB1, and HLA-DRB5. Allele-specific changes in expression were observed not only on MAPT but also on MAPT-AS1, CRHR1, KANSL1-AS1, LRRC37A2, MAPK8IP1P1, and MAPK8IP1P2. Regional association plots for the MAPT and HLA-DPB2 regions were generated using LocusZoom (Pruim et al., 2010), provided in Figure 3. Linkage data were provided by the International HapMap Project (The International HapMap Consortium, 2003). Supplementary Figures 8–10 provide genome-wide association and box-plots of gene expression by genotype for four MAPT and four HLA-region SNPs identified in eQTL analysis. Supplementary Spreadsheet S3 contains all significant eQTLs with SNP (rsID), location, and *p*-value.

### Perturbation Testing

To determine specific sources of genetic variation and their effects on the transcriptome, we overlapped all predicted eQTLs with all genes classified in co-expression modeling. Less than 1% of eQTLs (*N* = 5,392 gene/SNP pairs) across the 522 genes overlapped genes identified in co-expression network modules. We observed no discernable pattern in eQTLs and classified genes. Although observed differences in co-expression based on segregation are largely unexplained by individual eQTLs, functional ontology and transcription factor enrichment analysis provided polymorphisms in multiple genes sharing the transcription factor IRF motifs M08887 and M00972, which regulate many HLA genes.

## Discussion

Our analysis revealed several key functional domains and pathways through which systematic deregulation occurs in LOAD. Co-expressed transcripts, transcription factors, and genomic loci were statistically significant contributors to LOAD progression via deregulation along several immune system pathways. In segregation co-expression analysis, we observed a substantial reduction in the organizational structure of several well-preserved, functional modules, as indicated by fewer classified genes and lower intramodular connectivity in MCI and AD subjects as compared to controls. In the course of this experiment, we improved specificity in detecting functionally relevant co-expression modules in a complex disease through rigorous QC protocols and data reduction schemes, namely, module preservation testing. This is significant because co-expression analyses produce very large networks with dozens of modules. This much data can be cumbersome and difficult to interpret. Network module erosion and gene shedding were observed in the microglia (“magenta”), synaptic (“yellow”), and Hippo signaling (“blue”) modules.

We chose to make the “magenta” module the focus of this discussion based on two statistical facts: (1) it was the most statistically significant module in functional gene enrichment analysis (Table 1) and (2) this module has been observed before in a similar study. Zhang et al. (2013) identified a module (“light cyan”) containing 537 genes in the human prefrontal cortex which were highly associated (*p*-value = 2.1e-87) with the same immune-related GO terms. Remarkably, 98 “light cyan” genes overlapped our “magenta” module. Assuming a hypergeometric distribution, the probability of identifying the same 98 genes from a total gene pool of 20,000 is 3.54e-129. We initially hypothesized that co-expression analysis would reveal cell-specific expression modules. Co-expression segregation analysis allowed us to compare specific changes in network and module organization.

The “magenta” module contained genes such as ABI3, CD33, MS4A46, MS4A6S, TREM2, and TYROBP, which have been previously linked to AD through protein-coding mutations (Naj et al., 2011; Sims et al., 2017) and are all critical to microglial activation and response (Satoh et al., 2017). Microglia are the principal innate immune cells of the brain and ingest and degrade amyloid plaques (Koenigsknecht-Talboo and Landreth, 2005). Segregation analysis based on COGDX showed that CD33 and TREM2 were co-expressed in NCI and MCI subjects but not in AD subjects, and CD4 was only co-expressed in the MCI module. CD33 and CD4 are associated with reactive microglia and have been linked to AD (Griciuc et al., 2013). TREM2 is activated by ligand binding and increases Aß clearance through the apoptosis-related phosphatidylinositol-3 kinase (PI3K) signaling pathway, while activating CD33 attenuates the innate immune response and Aß clearance. CD33 and TREM2 showed high heteroskedasticity in AD subjects and have been suggested as cross-talking Alzheimer’s genes (Chan et al., 2015). Taken together, our data suggest that CD33 and TREM2 co-regulation are important to maintaining healthy brain aging. ANOVA analysis of the “magenta” module showed increased expression in many genes including IL10RA and CD37. CD37 is activated by Aβ and mediates both humoral and cellular immune responses (van Spriel et al., 2004, 2009). This module also included HLA-DMA, HLA-DMB, and HLA-DRA.

Since the purpose of this study was to compare normal brain aging with AD, underlying aging pathways were not directly assessed. However, we noticed an interesting overlap with previous findings in a WGCNA study on the aging of the prefrontal cortex (Hu et al., 2018). Hu et al. reported a module enriched in the synaptic vesicle cycle function associated with brain aging progression. This module (“blue”) overlaps with the enrichment of our “yellow” module defined here. Within the GO term “synaptic vesicle cycle,” seven genes (AP2M1, ATP6V0D1, DNM1, RAB3A, STX1A, UNC13A, and VAMP2), involved in vesicle transport, endocytosis, and exocytosis, are shared between both studies. The difference in platforms and sample sizes makes this similarity remarkable, suggesting a further manifestation of synaptic dysfunction and impaired cognition in LOAD. The “blue” module was significantly enriched for Hippo signaling, which not only has implications on cell growth and autophagy but also the immune system (Zhang et al., 2018). Aß has been shown to initiate nuclear pro-apoptotic transcription factors in the Hippo signaling pathway, resulting in neuronal death (Sanphui and Biswas, 2013).

Our second motivation for the study was to examine the common genetic variants associated with LOAD. LOAD is likely influenced by the interaction of many polygenic, low- and moderate-effect variants. In our study, less than 1% of eQTLs overlapped genes classified in co-expression modeling. Of course, this did not directly explain changes in coexpression; however, eQTL analysis provided perturbations in multiple, functionally related genes (HLA-A, HLA-C, HLA-DOB, HLA-DP1, HLA-DRB1, and HLA-DRB5), all sharing transcription factor motif IRF. Interferon-regulatory factors modulate the interferon system in innate and adaptive immunity, and INF-γ induces differential expression of MHC class II HLA-DR and HLA-DP genes (Helbig et al., 1991). IFN-γ is expressed by infiltrating Th1 cells, resident microglia, and neurons and has been implicated in the development of AD and systemic autoimmunity. IFN-γ signaling is known to adversely affect AD pathologies and cognitive function (Mastrangelo et al., 2009; Monteiro et al., 2016). Activation of microglia by INF-γ inhibits Aβ clearance (Bate et al., 2006; Browne et al., 2013). HLA region eQTLs and changes in IFN-γ signaling can partially explain transcriptomic immune deregulation observed in cases.

The high concentration of eQTLs in the MAPT region highlights the impact of genetic variation on disease risk not only through MAPT haplotypes but also in several neighboring genes. MAPT pathologies provide a mechanistic link between the immune system and neurodegeneration involving microglia activation (Bhaskar et al., 2010). Splice-variants of MAPT-AS1 actively suppress MAPT translation (Coupland et al., 2016) and could prove to be a useful therapeutic target by reducing hyperphosphorylated tau levels. KANSL1 is critical to brain development (Koolen et al., 1993) and has been linked to AD (Jun et al., 2016). Corticotropin Releasing Hormone Receptor 1 (CRHR1) agonists have been shown to increase Aβ production (Futch et al., 2017). Determining the precise nature of the relationship between genetic variation and the expression of MAPT region genes will undoubtedly provide additional insights into tauopathy and thus LOAD risk.

Immune network architectures account for desirable immune system properties such as inducibility, adaptability, and robustness (Schrom et al., 2017). Data segregation combined with co-expression analysis sheds light onto these processes in LOAD, revealing adaptations during disease onset and erosion of networks in the later stages. Observed increases in transcriptional heterogeneity resemble observations in Parkinson’s disease (Mar et al., 2011), but can only partially account for module erosion since many highly variable genes are still present in the AD modules. Taken together, this study provides insights into a complex and dynamic landscape of genetic and regulatory processes centered around innate and adaptive immune system function. Systematic reductions in co-regulated genes and intramodular connectivity were diagnostic of increasing variability in several critical LOAD pathologies, including neuroinflammation, adaptive immunity, synaptic loss, and apoptosis. We propose that a reduction in regulatory and compensatory systems could also account for decreased robustness during disease progression, but the underlying mechanisms and the combined role of genetic variants are far from clear. This study highlights the adequacy of combining multi-omics NGS data types with longitudinal clinical and other developing, deep-phenotype data to decipher the complex molecular dynamics underlying complex diseases like LOAD.

## Data Availability Statement

All data analyzed were obtained from the Accelerating Medicines Partnership for Alzheimer’s Disease (AMP-AD) Data Portal and can be accessed at https://www.synapse.org/#!Synapse:syn2580853/wiki/409844.

## Author Contributions

JM performed co-expression, functional, GSEA, and eQTL analyses. AK performed TF analysis and supervised the study. JM and AK analyzed the functional outcomes and wrote the manuscript.

## Conflict of Interest

The authors declare that the research was conducted in the absence of any commercial or financial relationships that could be construed as a potential conflict of interest.
