# Supplementary material for: Erosion of Gene Co-expression Networks Reveal Deregulation of Immune System Processes in Late-Onset Alzheimer’s Disease
Source: Front Neurosci. 2020 Mar 20;14:228. doi: 10.3389/fnins.2020.00228 (PMC7099620; doi:10.3389/fnins.2020.00228)
Supplement: Supplementary file 1 [file Data_Sheet_1.pdf]

Supplementary Material

Erosion of Gene Co-expression Networks Reveal Deregulation of Immune System Processes in late-onset Alzheimer's Disease

John Stephen Malamon[1], Andres Kriete[1]

[1]Bossone Research Center, School of Biomedical Engineering, Science and Health Systems, Drexel University, Bossone Research Center, 3141 Chestnut Street, Philadelphia, PA 19104, USA.

1 Supplementary Data

This supplement outlines the methodological details and results for the joint analysis of DNA-seq, RNA-seq, and clinical neuropathological (NP) data from 503 human subjects (305 cases/198 controls) with an elevate risk for late-onset Alzheimer's disease (LOAD). Subjects ranged from greater than 67 to 90 years of age. Ages above 90 are reported as "+90". Subjects includes 322 males and 181 females with average Braak, CERAD, and COGDX scores of 3.5049, 2.3339, and 2.592, respectively. Eleven subjects showed mixed pathologies (COGDX=6) and were included as cases in all analyses. All subjects reported race as Caucasian and were randomly sampled for APOE genotype. All methodological details should be sufficient for the replication of this study. Please contact the corresponding author for additional information. All NGS data are publicly available at the AMP-AD Knowledge Portal at https://www.synapse.org/#!Synapse:syn5550382.

1.1 Analysis Framework

Our framework for the combined analysis of genotype, gene expression, and clinical data is outlined in *Supplementary Figure 1*. The four vertical columns represent the four main phases of analysis: quality control (QC), network, functional, and perturbation analysis. All RNA-seq data were passed through a comprehensive QC protocol, which is outlined in the next section. The QC'd RNA-seq data were then used for network construction using WGCNA. Network modules were classified and compared to clinical eigenvectors to discover associations between modules and clinical traits. Functional enrichment using GO was applied to identify statistically significant modules that were associated with known LOAD pathologies. Next, we applied WGCNA's module preservation testing to only keep well-preserved (Z-score > 10) and reproducible modules. These functional and reproducible modules become candidate pathways. To replicate associated transcripts and pathways discovered using our framework, we applied the Broad's gene set enrichment (GSEA) toolkit. Finally, perturbation testing was performed by overlapping genes discovered in network analysis with expression quantitative trait loci (eQTLs) to explain changes in transcriptomic organization.

1.2 RNA-seq Quality Control

There are many sources of heterogeneity in these data, rooted in the preparation of macro-dissected tissue as well as in the contributions of different cell types with varying levels of dysfunction. We have applied a comprehensive quality control and data reduction protocol, including log transformation, entropy and connectivity filtering, batch effect correction, and sample-level normalization. For entropy filtering, we calculated Shannon-Wiener's Index of Diversity to measure

the information density of each transcript. We excluded all transcripts below the first quartile or 2.5 bits. Next, we used WGCNA to calculate the connectivity for all transcripts in the network and remove approximately 8,000 transcripts with the fewest network connections (number of degrees). Degrees represent the number of associations (edges) between network eigengenes. After entropy and connectivity filtering, we proceeded with the 20,000 most informationally dense and connected transcripts. We used ComBat to normalize the expression data and adjust for the two sequencing batches (ROS and MAP). For sample normalization, we applied "Trimmed Mean of *M*-values" or TMM normalization to all samples and adjusted expression values.

1.3 Clinical Principal Components Analysis

We initially performed a principal components analysis (PCA) on all clinical NP data. *Supplementary Figure 2* provides a bi-directional Euclidian plot or bi-plot for the 1st (PC1) and 2nd (PC2) PCs for all individuals. The 1st and 2nd PCs accounted for just over 50% of the observed variance. COGDX, Braak and disease status moved in the same direction, while CERAD moved in the opposite direction. This is because CERAD is coded in the opposite direction as the other metrics. For example, '1' is the diseased phenotype and '4' the normal phenotype, whereas with COGDX '1' is the normal phenotype and '4' and '5' are diseased. In summary, the clinical NP data consistently agree across all subjects, whilst sex and APOE genotype add variation to the second PC.

1.4 ANOVA of mRNA Levels by COGDX Subgroups

Because the 'magenta', 'yellow', and 'blue' modules exhibited high preservation Z-scores, functional relevance to known LOAD pathologies, and topological erosion (gene and connectivity loss), we applied one-way ANOVA and Bartlett's test for heteroscedasticity between all mRNA levels for three the COGDX clinical subgroups (NCI, MCI, and AD). ANOVA and Bartlett's test for heteroscedasticity were performed for all transcripts by COGDX subgroup revealing a significant (p-value < 0.05) increase in the expression of 22 'magenta', 31 'yellow', and 70 'blue' genes. Heteroscedasticity was significant (p-value < 0.001) for 36, 48, and 74 genes, respectively. These data provide supporting evidence for the deregulation of gene networks in these three modules. *Supplementary Spreadsheet 2* contains all ANOVA and Bartlett's testing results. These data suggest systematic deregulation via significantly increased variability and alternations in expression levels in association with COGDX.

1.5 Cell-type Enrichment for Seven Reproducible Module

To examine cell-specific contributions in reproducible co-expression modules, we queried the Human Protein Atlas and ARCHS4 Tissues database using the Enrichr website. Both databases offer high-quality and high-volume cellular and tissue-level RNA expression profiles. All results are provided in *Supplementary Table 2.* Identifying cell-types in bulk tissue-derived RNA is not the aim of this study; however, we originally hypothesized that specific cell-type signatures would be present in co-expression modules. Therefore, we queried genes contained in the seven modules that passed preservation testing. The 'magenta' module is significant for 'Monocytes', whereas other modules like 'blue' and 'brown" are significant for 'Whole Brain'.

2 Supplementary Tables and Figures

2.1 Supplementary Tables

Supplementary Table 1. Description of ROSMAP clinical neuropathology data. This table provides a general description for all clinical neuropathology data used in this study. Please see the AMP-AD Knowledge Portal for more information.

Trait	Values	Description
Braak stage	0 – 6	Progression of neurofibrillary tangles (NFTs)
CERAD score	1 – 4	Semiquantitative assessment of neuritic plaque:<br>1 – Definite<br>2 – Probable<br>3 – Possible<br>4 – No AD
COGDX score	1-6	Final cognitive diagnosis:<br>1 – No cognitive impairment (NCI)<br>2,3 – Mild cognitive impairment (MCI)<br>4,5 – Alzheimer’s diagnosis (AD)<br>6 – Other dementia
Disease status	0 or 1	0 – Control, 1 – Case

Supplementary Table 2. Cell-type enrichment using the Human Gene Atlas and ARCHS4 Tissue databases. EnrichR results for Human Protein Atlas and ARCHS4 Tissue database queries.

Module Name	Human Protein Atlas	p-value	ARCHS4 Tissues	p-value
Magenta	CD14+ Monocytes	1.623E-53	MACROPHAGE	3.597E-91
Yellow	CD19+ BCells	0.0003642	NEURONAL EPITHELIUM	2.759E-20
Blue	Whole Brain	7.185E-26	SUPERIOR FRONTAL GYRUS	1.607E-90
Turquoise	Amygdala	5.733E-24	CINGULATE GYRUS	6.065E-58
Green	Whole-blood	0.001366	Not Significant	N/A
Red	CD19+ BCells	0.000388	PLACENTA (BULK)	0.001566
Brown	Whole Brain	2.376E-25	SUPERIOR FRONTAL GYRUS	4.417E62

2.2 Supplementary Figures

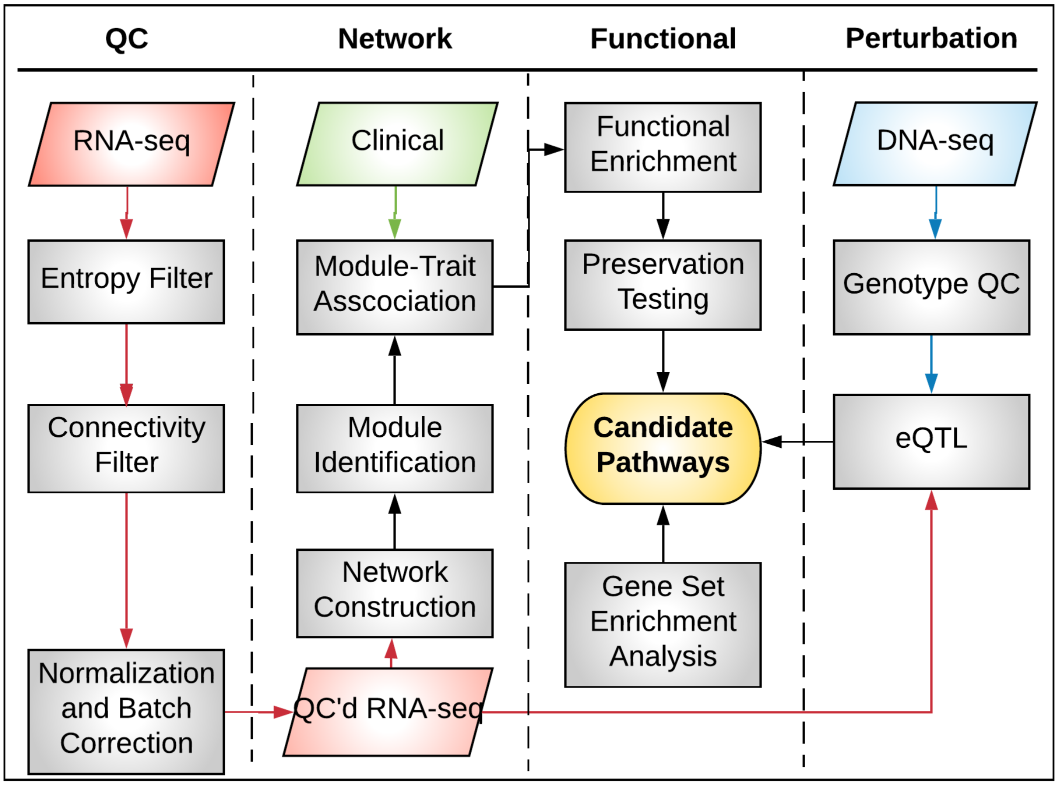

Supplementary Figure 1. Analytical framework overview. This image provides an overview of our analysis workflow for integrating genotype, gene expression, and clinical data. The four main analytical components (QC, Network, Functional, and Perturbation) are shown in each lane (grey boxes) with each processing step for the three data types: RNA-seq (red), clinical data (green), and DNA-seq (blue). Network analysis combines post-QC RNA-seq data with clinical data. Functional analyses include ontology enrichment and gene set enrichment analysis. Perturbation testing overlaps eQTLs with co-expressed genes discovered in the Network phase.

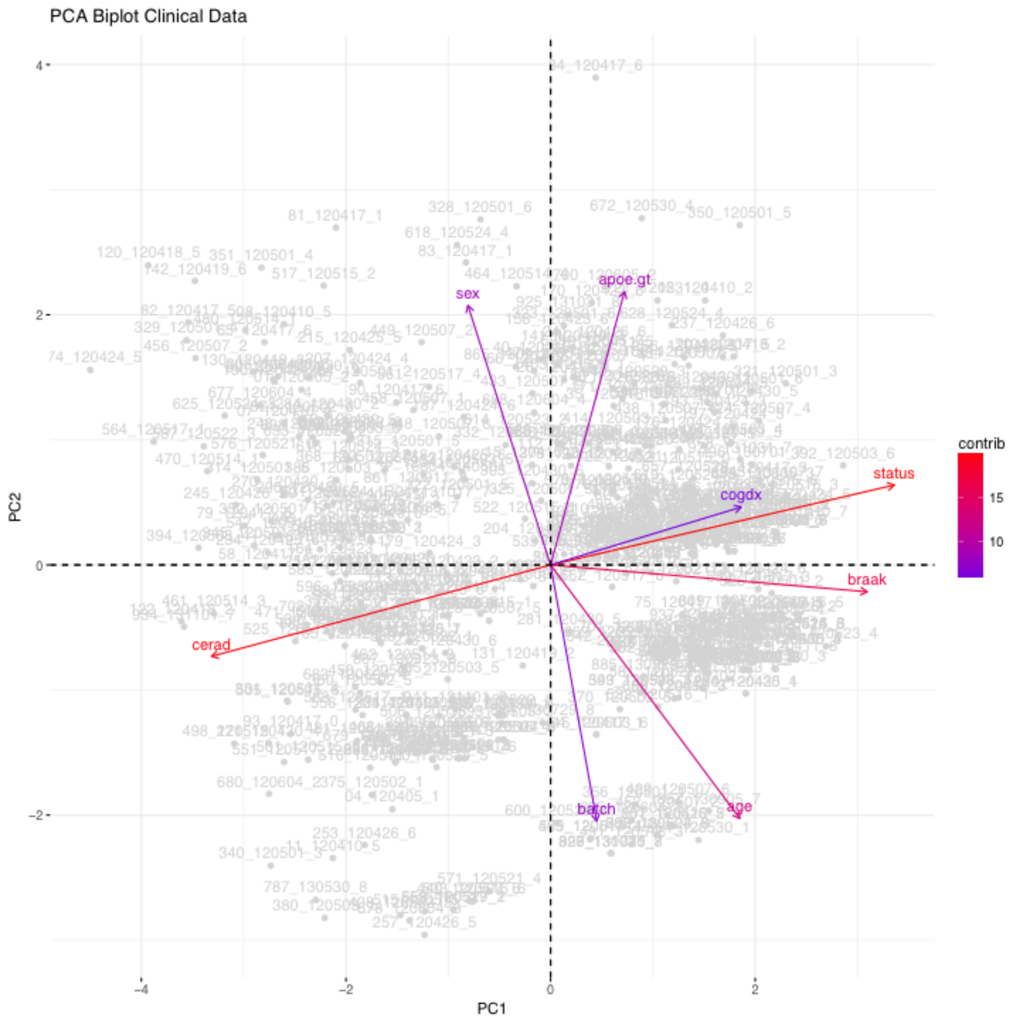

Supplementary Figure 2. PCA bi-plot of all clinical ROSMAP data. 1st PC versus 2nd PC for all clinical information including neuropathology metrics (CERAD, COGDX, Braak staging, and status) as well as age, sex, and APOE genotype (apoe_gt) for all 503 subjects (305 cases/198 controls). The 'contrib' value (color map legend) provides the estimated contribution to variation in the PCs for each clinical variable.

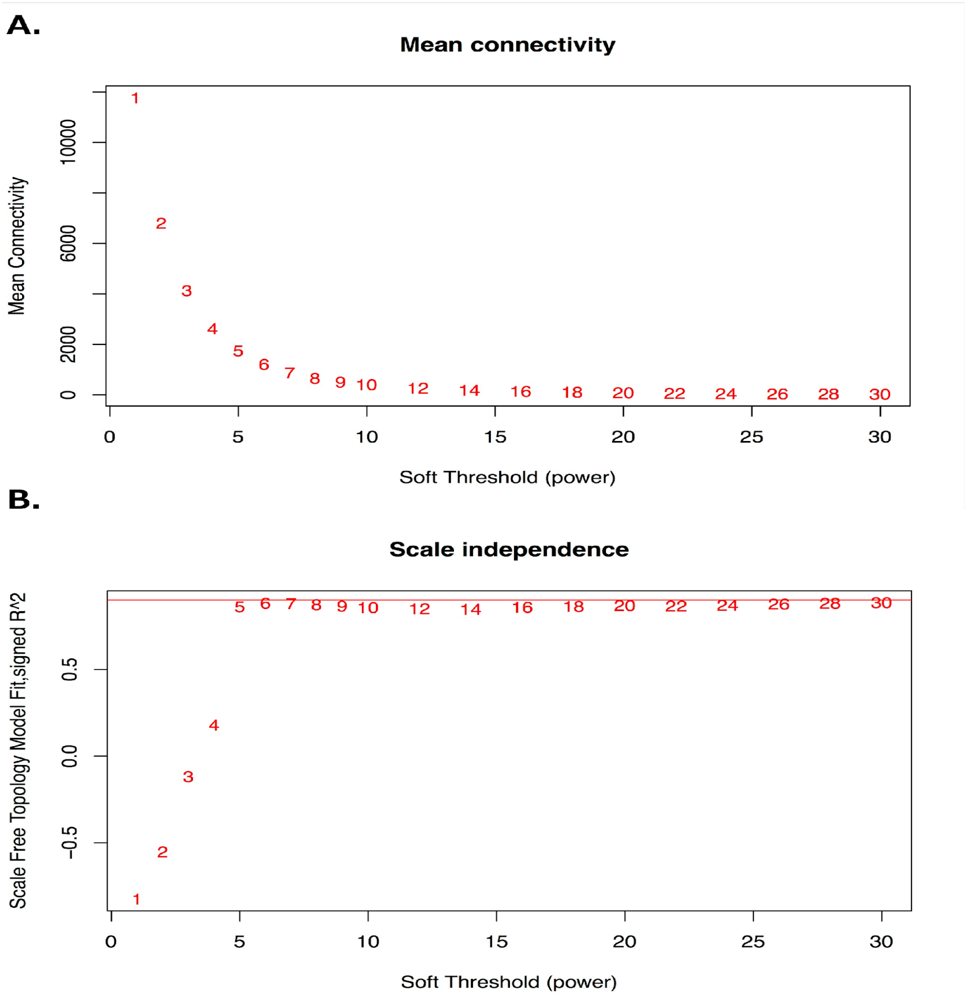

Supplementary Figure 3. Connectivity and SFT plots for transcriptomic network. A) shows the mean network connectivity (degrees) as a function of the power or number of correlation iterations and B) provides R^2 values as a function of the soft-threshold power. Red line is set at 0.85.

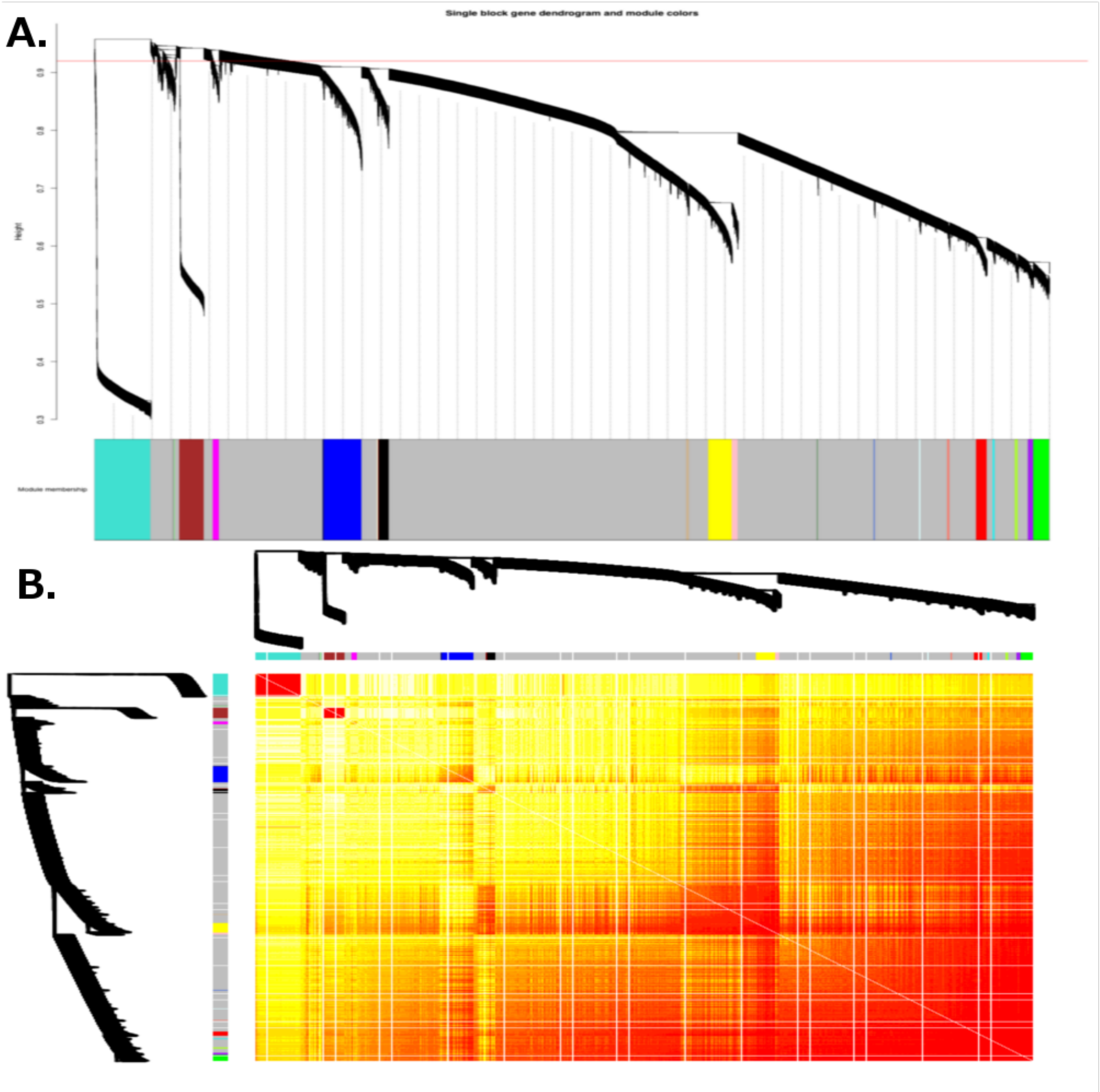

Supplementary Figure 4. Dendrogram and heatmap of transcriptomic network. A) Gene module groupings as a dendrogram with dissimilarity plotted vertically. Cut height was set at 0.92, as indicated by the red line. Genes in the grey bands of the bottom lane are not assigned to modules. B) Heatmap of similarity (red) across transcriptome network. We identified 26 distinct modules, totaling 4,429 transcripts with an average of 201 genes per module.

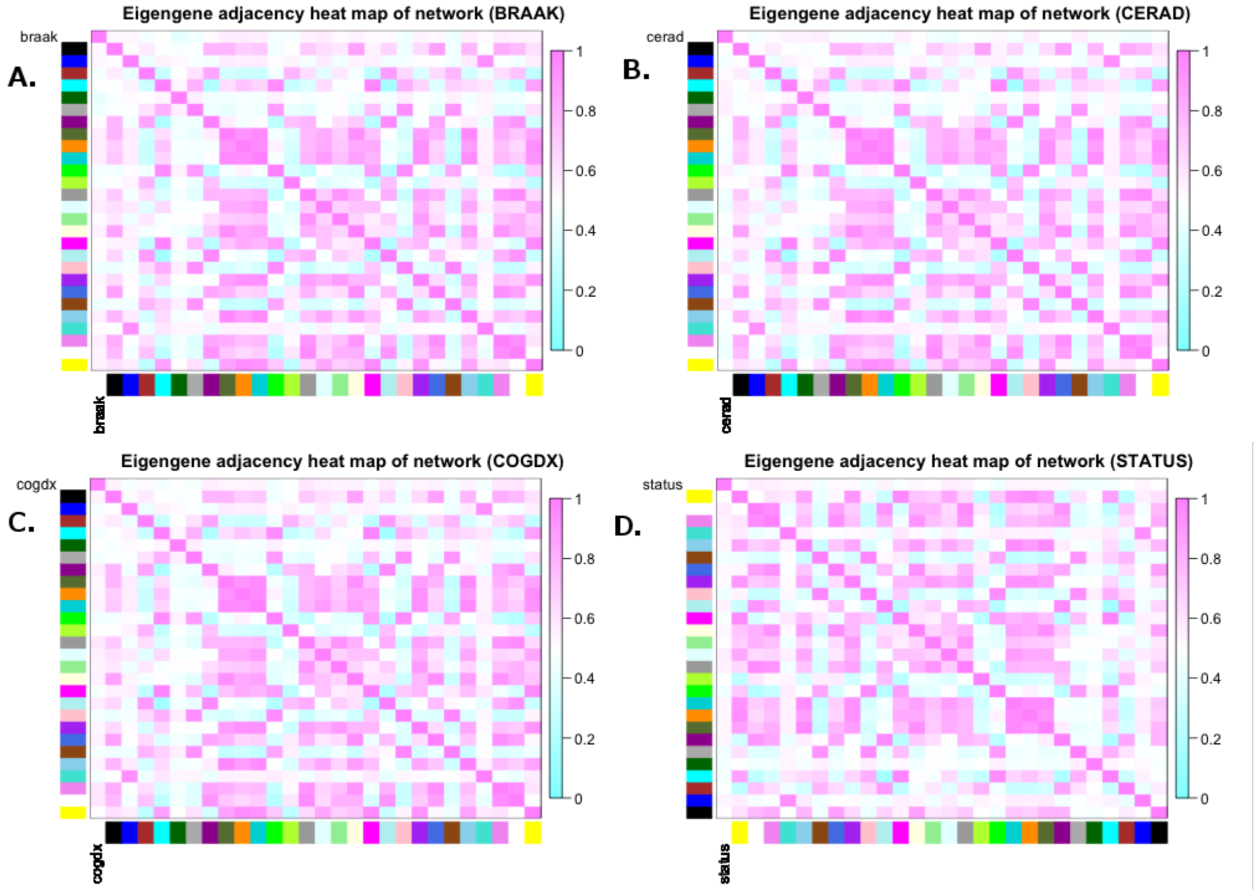

Supplementary Figure 5. Heatmap of correlations for all module eigengenes and four clinical NP traits. Eigengene-to-eigengene and eigengene-to-trait correlations for all modules and four clinical traits: Braak (A), COGDX (B), CERAD (C), and disease status (D). The associated clinical trait is labeled on each axes.

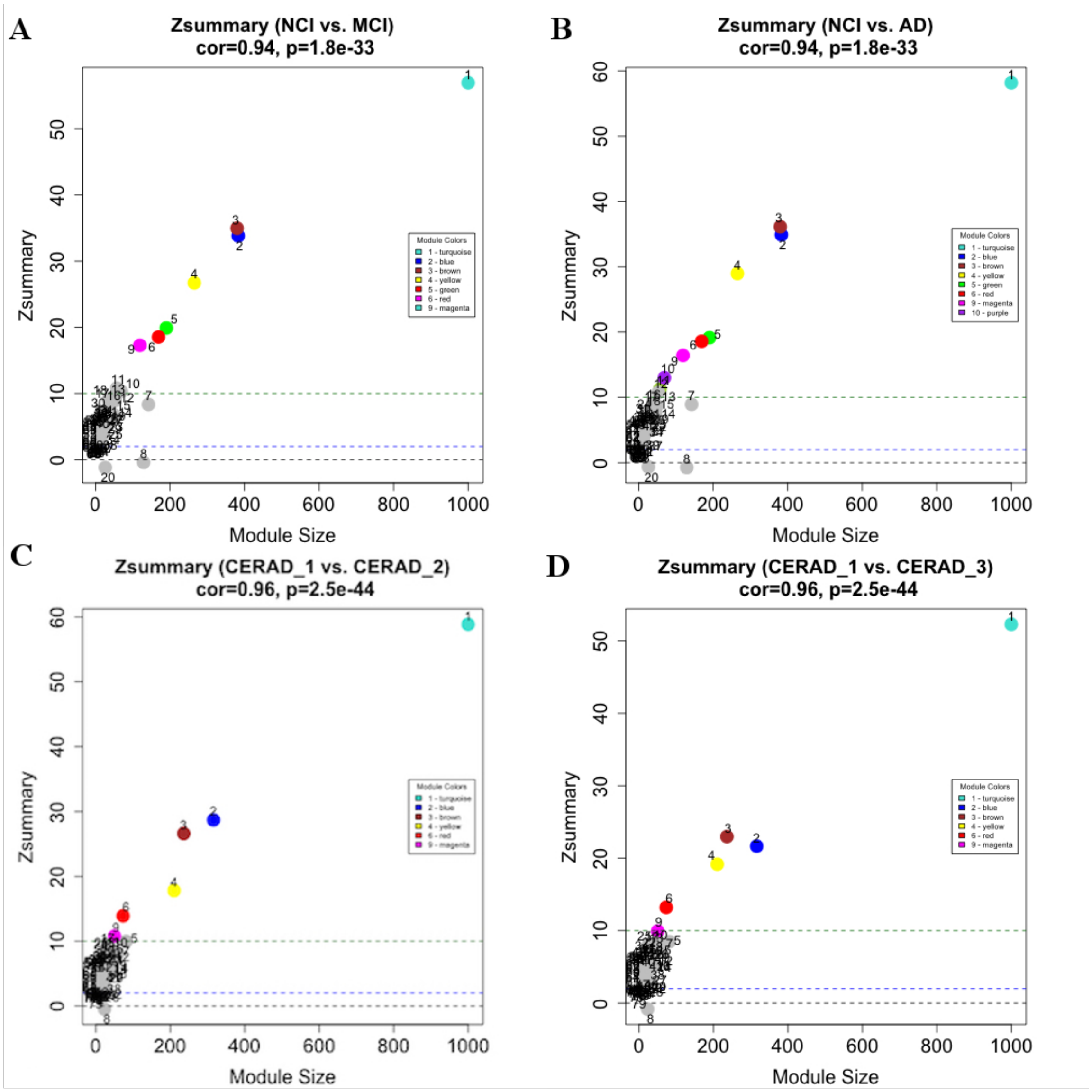

Supplementary Figure 6. Module preservation Z-score testing for segregated neworks. A) Z-scores plotted by module size for NCI versus MCI group (COGDX); B) Z-scores plotted by module size for NCI versus AD subgroup (COGDX); C) Z-scores plotted by module size for CERAD_1 versus CERAD_2 subgroup; and D) Z-scores plotted by module size for CERAD_1 versus CERAD_3 subgroup. The dotted green line ('Zsummary'=10) indicates significantly preserved modules. 'Zsummary' and module size are highly correlated at 0.94 and 0.96 for COGDX and CERAD. P-values are provided by Pearson's methods. Each module is labeled by the ordinal rank of its size. Segregation using COGDX shows increased reproducibility and stability in module preservation over segregation based on plaque (CERAD) assessment scores.

Pathway	Gene ranks	NES	pval	padj
KEGG_CELL_ADHESION_MOLECULES_CAMS		2.11	2.1e-05	1.5e-03
KEGG_LEISHMANIA_INFECTION		2.24	4.2e-05	1.5e-03
KEGG_ANTIGEN_PROCESSING_AND_PRESENTATION		2.13	4.2e-05	1.5e-03
KEGG_SYSTEMIC_LUPUS_ERYTHEMATOSUS		2.27	4.2e-05	1.5e-03
KEGG_OLFACTORY_TRANSDUCTION		1.65	1.6e-04	3.1e-03
KEGG_ALZHEIMERS_DISEASE		-1.70	9.5e-04	9.9e-03
KEGG_CARDIAC_MUSCLE_CONTRACTION		-2.07	1.2e-04	2.5e-03
KEGG_PARKINSONS_DISEASE		-1.99	7.6e-05	1.9e-03
KEGG_HUNTINGTONS_DISEASE		-1.86	7.4e-05	1.9e-03
KEGG_OXIDATIVE_PHOSPHORYLATION		-2.20	1.9e-05	1.5e-03

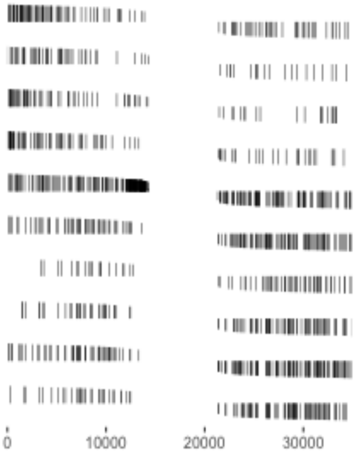

Supplementary Figure 7. Table of top 10 KEGG pathways from GSEA analysis. Top five pathways sorted by p-value associated with cases (top five) and controls (bottom five) for gene set enrichment analysis. Negative enrichment scores denote an over-representation of pathway gene expression in cases. The third column provides the normalized enrichment score.

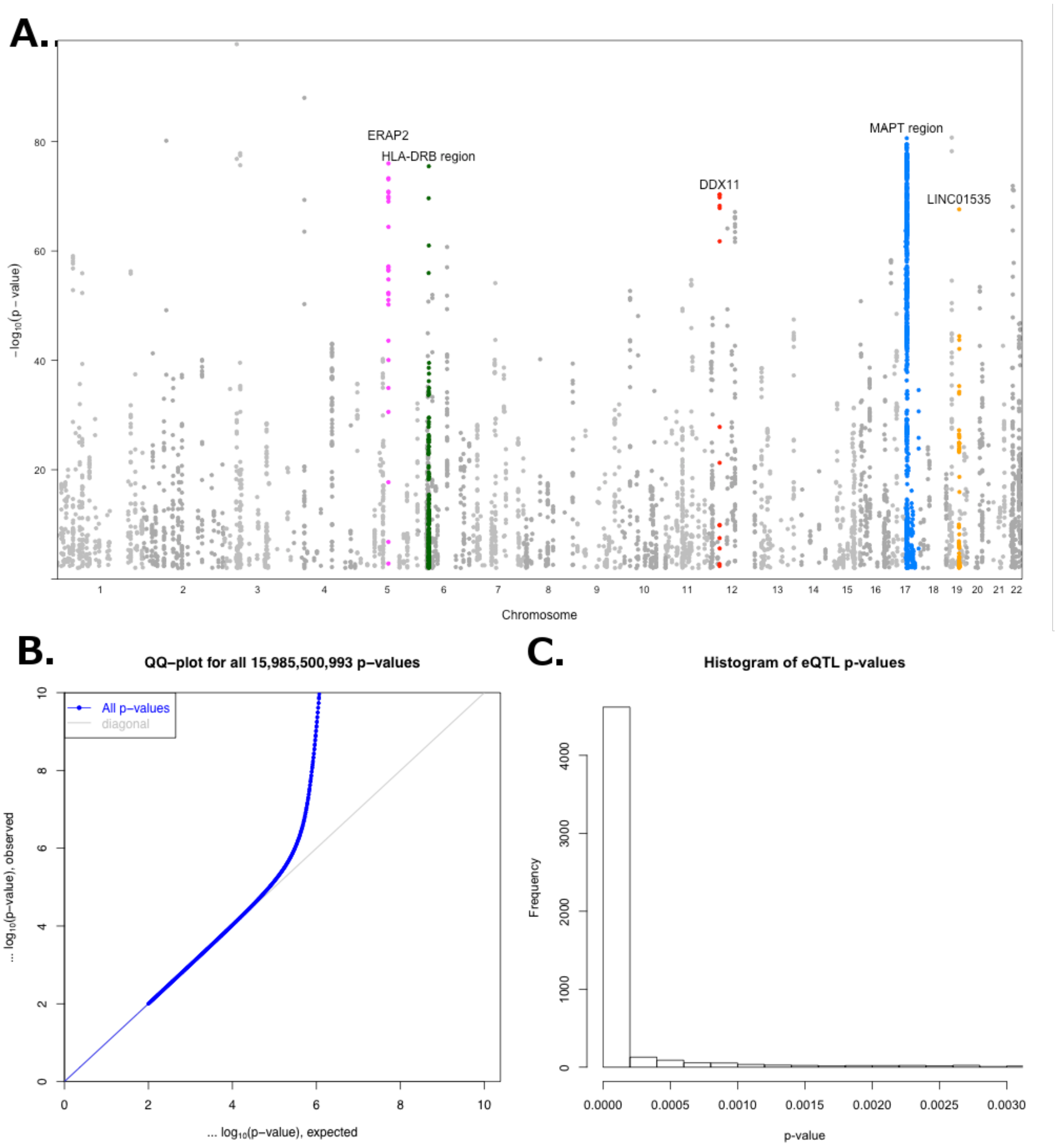

Supplementary Figure 8. Genome-wide association plots. A) Association p-values (-$\log_{10}$ scale) for all autosomal eQTLs above the significant threshold of 0.01 (N=5,392), B) quantile-quantile plot of observed versus expected eQTL p-values, and C) histogram of all eQTL p-values.

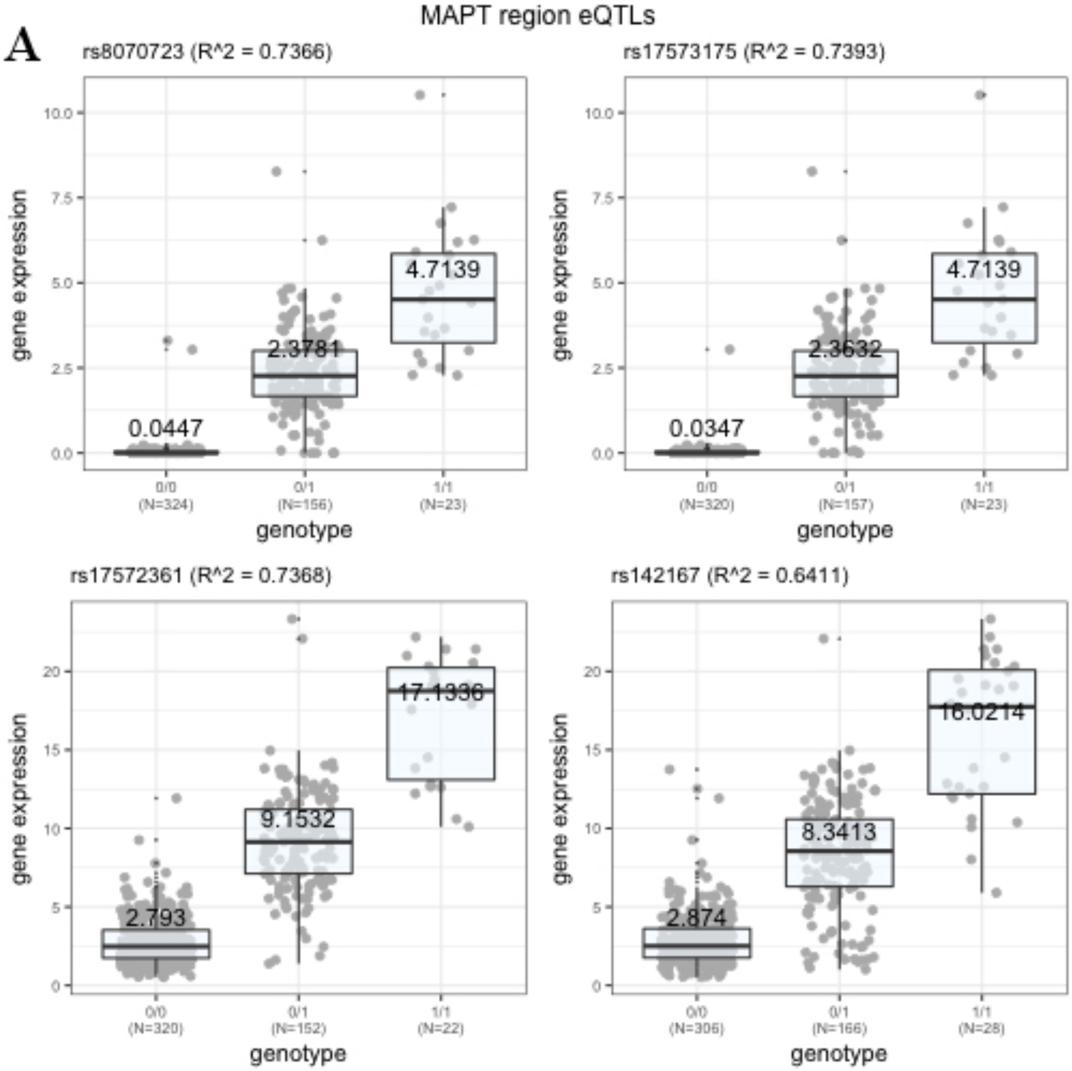

Supplementary Figure 9. Gene expression plotted by genotype for four cis eQTLs (SNP/gene pairs) on the MAPT region. Top and bottom SNP pairs are predicted to affect MAPK8IP1P1 and KANSL1-AS1, respectively.

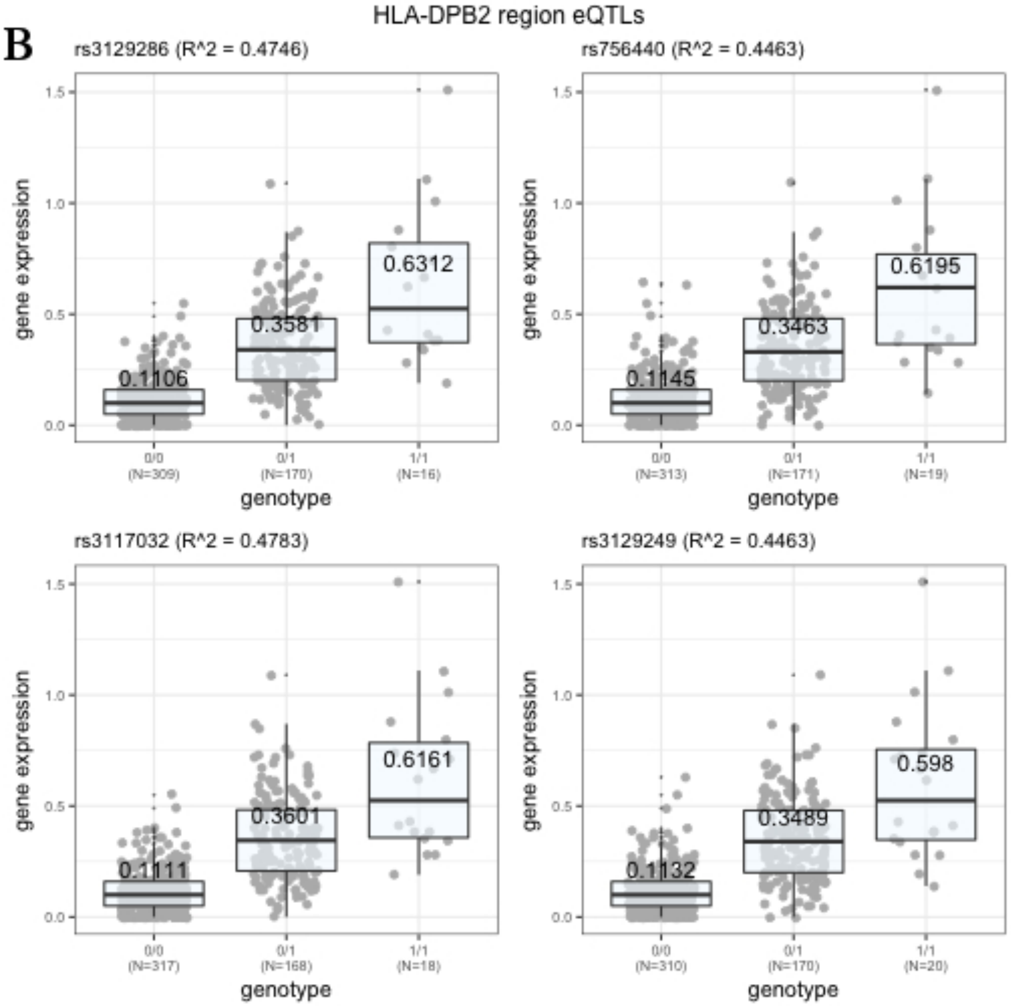

Supplementary Figure 10. Gene expression plotted by genotype for four cis eQTLs (SNP/gene pairs) on the HLA region. Top and bottom SNV pairs are predicted to the gene expression of HLA-DPB2 plotted by genotype for four cis eQTLs.
